# No impact of attenuation and scatter correction on the interpretation of dopamine transporter SPECT in patients with clinically uncertain parkinsonian syndrome

**DOI:** 10.1007/s00259-023-06293-2

**Published:** 2023-06-17

**Authors:** Tassilo Schiebler, Ivayla Apostolova, Franziska Lara Mathies, Catharina Lange, Susanne Klutmann, Ralph Buchert

**Affiliations:** 1 Department of Diagnostic and Interventional Radiology and Nuclear Medicine, University Medical Center Hamburg-Eppendorf, Martinistr, 52, 20246 Hamburg, Germany; 2 Department of Nuclear Medicine, Charité - Universitätsmedizin Berlin, Corporate Member of Freie Universität Berlin and Humboldt-Universität Zu Berlin, Berlin, Germany

**Keywords:** Dopamine transporter, SPECT, Ioflupane, ^123^I-FP-CIT, Attenuation correction, Scatter correction

## Abstract

**Purpose:**

The benefit from attenuation and scatter correction (ASC) of dopamine transporter (DAT)-SPECT for the detection of nigrostriatal degeneration in clinical routine is still a matter of debate. The current study evaluated the impact of ASC on visual interpretation and semi-quantitative analysis of DAT-SPECT in a large patient sample.

**Methods:**

One thousand seven hundred forty consecutive DAT-SPECT with ^123^I-FP-CIT from clinical routine were included retrospectively. SPECT images were reconstructed iteratively without and with ASC. Attenuation correction was based on uniform attenuation maps, scatter correction on simulation. All SPECT images were categorized with respect to the presence versus the absence of Parkinson-typical reduction of striatal ^123^I-FP-CIT uptake by three independent readers. Image reading was performed twice to assess intra-reader variability. The specific ^123^I-FP-CIT binding ratio (SBR) was used for automatic categorization, separately with and without ASC.

**Results:**

The mean proportion of cases with discrepant categorization by the same reader between the two reading sessions was practically the same without and with ASC, about 2.2%. The proportion of DAT-SPECT with discrepant categorization without versus with ASC by the same reader was 1.66% ± 0.50% (1.09–1.95%), not exceeding the benchmark of 2.2% from intra-reader variability. This also applied to automatic categorization of the DAT-SPECT images based on the putamen SBR (1.78% discrepant cases between without versus with ASC).

**Conclusion:**

Given the large sample size, the current findings provide strong evidence against a relevant impact of ASC with uniform attenuation and simulation-based scatter correction on the clinical utility of DAT-SPECT to detect nigrostriatal degeneration in patients with clinically uncertain parkinsonian syndrome.

**Supplementary information:**

The online version contains supplementary material available at 10.1007/s00259-023-06293-2.

## Introduction 

Single photon emission computed tomography (SPECT) of striatal dopamine transporter (DAT) availability with N-ω-fluoropropyl-2β-carbomethoxy-3β-(4-I-123-iodophenyl)nortropane (^123^I-FP-CIT) is widely used to support the diagnostic work-up in patients with a clinically uncertain parkinsonian syndrome (CUPS) [1–5].

The accuracy of SPECT for quantitative imaging of the spatial radioactivity distribution is affected by physical effects including attenuation, scatter and septal penetration of photons. Photon attenuation causes a reduction of the striatal signal by approximately 70% in DAT-SPECT [6]. Photon scatter results in mislocation of striatal counts into extrastriatal brain regions and, therefore, causes further reduction of the contrast between the striatum and extrastriatal brain regions [7]. Furthermore, ^123^I emits about 2.5% high-energy (≥ 440 keV) photons that cause septal penetration (and scatter) counts in the standard energy window centered at 159 keV [8]. Septal penetration is not restricted to photons originating from the brain, but high uptake and retention of ^123^I-FP-CIT in the lungs, liver, intestines, salivary glands, and thyroid also contaminate the brain image through septal penetration [9, 10]. The effect of scatter and septal penetration combined is larger than the effect of photon attenuation under most circumstances [10].

Attenuation correction and scatter correction methods are available to reduce the deterioration of the quantitative image quality by these physical effects [11–13]. Scatter correction usually reduces the impact of septal penetration, too [14]. The combination of attenuation and scatter correction (ASC) typically results in about 50% increase of the striatal ^123^I-FP-CIT specific binding ratio (SBR) [10, 15–17]. Thus, ASC is considered mandatory for quantitative characterization of the “true” striatal DAT-availability as a biomarker in research [10, 14, 18].

The clinical utility of ASC in DAT-SPECT for the differentiation between neurodegenerative (due to nigrostriatal degeneration) and non-neurodegenerative (secondary) Parkinsonian syndromes is less clear, and DAT-SPECT is performed without these corrections at many sites [10, 19]. In a relatively small sample of 77 DAT-SPECT scans, the overall accuracy of the putaminal ^123^I-FP-CIT SBR for the detection of neurodegenerative parkinsonian syndromes was not affected by ASC (area under the receiver operating characteristic curve 0.921 without ASC versus 0.919 with ASC), but ASC resulted in somewhat higher sensitivity (83.7% versus 76.7%) at somewhat lower specificity (85.3% versus 91.2%) [14].

The benefit of ASC for visual interpretation of DAT-SPECT images is even less clear. Some procedure guidelines for DAT-SPECT state that ASC does not necessarily benefit the visual interpretation of the SPECT images [1]. Other guidelines consider the necessity and the required accuracy of ASC for clinical DAT-SPECT an open question [20].

Against this background, the current study evaluated the impact of ASC on both semi-quantitative analysis and visual interpretation in a large sample of 1740 DAT-SPECT. Attenuation correction was based on uniform attenuation maps, scatter correction on simulation.

## Materials and methods

### Patients

The picture archiving and communication system of our institution was searched for DAT-SPECT with ^123^I-FP-CIT that had been performed due to a clinically uncertain suspicion of nigrostriatal degeneration. The only inclusion criteria were that SPECT had been performed with low-energy-high-resolution parallel-hole or fan-beam collimators and that the raw projection data were available for consistent retrospective image reconstruction. This identified 1765 DAT-SPECT. The corresponding DAT-SPECT images were inspected visually to exclude cases with atypical reduction of striatal ^123^I-FP-CIT uptake most likely caused by structural/vascular lesions. This led to the exclusion of 25 of the 1765 DAT-SPECT. The remaining 1740 DAT-SPECT from 1712 different patients (43.3% females) were included. The mean age at the time of DAT-SPECT was 66.7 ± 11.6 years (range 20.2–90.8 years). There were no further eligibility criteria to make sure that the included data were representative of everyday clinical routine.

### SPECT imaging

DAT-SPECT had been performed between December 2008 and January 2020 according to common procedure guidelines [21–23] with four different hardware settings: Siemens e.cam (Siemens Healthineers, Erlangen, Germany) dual-head camera equipped with low-energy-high-resolution collimators (*n* = 704), Siemens Symbia TruePoint dual-head camera with low-energy-high-resolution (*n* = 147) or fan-beam collimators (*n* = 457), and Mediso AnyScan Trio (Mediso, Budapest, Hungary) triple-head camera equipped with low-energy-high-resolution-high-sensitivity collimators in dual-head mode (*n* = 432). Detailed acquisition parameters are given in Supplementary Table 1.

In order to reduce variability of no interest associated with the different hardware settings and/or the use of different reconstruction algorithms over time, all raw projection data were reconstructed retrospectively using iterative ordered-subsets-expectation–maximization [24] with attenuation and simulation-based scatter correction as well as collimator-detector response modeling as implemented in the HybridRecon-Neurology tool of the Hermes SMART workstation v1.6 (Hermes Medical Solutions, Stockholm, Sweden) [25–28]. All parameter settings were as recommended by Hermes [25] for the EANM/EANM Research Ltd (EARL) ENC-DAT project (European Normal Control Database of DaTSCAN) [10, 16, 29–31]. More precisely, ordered-subsets-expectation–maximization was performed with 5 iterations and 15/16 subsets for 120/128 views. For noise suppression, reconstructed images were postfiltered by convolution with a 3-dimensional Gaussian kernel of 7 mm full-width-at-half-maximum. The simulator of the SPECT-acquisition process has been described in [26, 27]. In brief, the simulator is composed of two components, a Monte Carlo simulator to track the photons in the patient and a collimator-detector response model. In the current study, the collimator-detector response was modeled by a Gaussian point-spread-function model (rather than using a separate Monte Carlo simulator) [26, 27]. The Gaussian model is based on collimator specifications and on the intrinsic spatial resolution of the detector (both to be provided by the user; Supplementary Table 1). The Monte Carlo simulator modeled scatter in the patient using the convolution-based forced detection algorithm simulating 10^5^ photons with 2 scatter update iterations [26, 27]. Attenuation was modeled using a uniform attenuation map with a narrow-beam attenuation coefficient of 0.146/cm and assuming exponential loss of radiation intensity according to Chang [25, 32]. The outer contour of the head for modeling of attenuation (and scatter) was delineated using the automatic thresholding-based approach implemented in the Hermes SMART workstation. This algorithm smoothes the non-corrected image by convolution with a 3-dimensonal isotropic Gaussian kernel of 25 mm full-width-at-half-maximum and then fits an ellipse to the 30% isocontour, separately in each transaxial plane [25]. The automatic delineation avoids variability of no interest associated with manual delineation. After all photons have been simulated, the subprojection maps are convolved with the collimator–detector response function from the Gaussian point-spread-function model.

Each SPECT scan was reconstructed also without ASC but otherwise exactly the same parameter settings as with ASC. In particular, collimator-detector response modeling was turned on in both cases. The batch utility of the HybridRecon-Neurology tool was used to reconstruct all 3480 (= 2*1740) images in a single batch in order to avoid errors by incorrect manual operation.

The rationale for using the same iterative ordered-subsets-expectation–maximization algorithm for image reconstruction with and without ASC was to avoid “contamination” of ASC effects with algorithm effects associated with the use of different reconstruction algorithms with and without ASC (e.g., filtered backprojection without ASC versus iterative ordered-subsets-expectation–maximization with ASC).

### Image preprocessing

Individual DAT-SPECT images were stereotactically normalized to the anatomical space of the Montreal Neurological Institute (MNI) using the normalize tool of the Statistical Parametric Mapping software package (version SPM12) with the following parameter settings: affine transformation (no nonlinear warping), template weighting by a binary mask of the whole head including the scalp and excluding the cerebellum, source weighting by a uniform cube of 200 mm edge length centered at the center of mass of the individual DAT-SPECT image in patient space, MNI template regularization, preserve concentrations, voxel size 2 × 2 × 2 mm^3^, trilinear interpolation, and no wrapping. Multiple custom DAT-SPECT templates representative of normal and different levels of Parkinson-typical reduction of striatal ^123^I-FP-CIT uptake were used as target for stereotactical normalization. SPM then finds the linear combination of these templates that best matches the patient’s image.

For each of the 1740 DAT-SPECT, stereotactical normalization was performed first for the image with ASC. The corresponding image without ASC was coregistered to the non-normalized ASC image and then stereotactically normalized using the same transformation as for the ASC image. The rationale for this was to avoid bias that might have been caused by independent stereotactical normalization of DAT-SPECT images with and without ASC.

Intensity scaling was achieved by dividing the voxel intensities of the stereotatically normalized images by the individual 75th percentile of the voxel intensity in a reference region comprising the whole brain without striata, thalamus, brainstem, cerebellum, and ventricles [33]. A binary mask of the reference region predefined in MNI space was used for this purpose. The scaled images are semi-quantitative images representing the distribution volume ratio (DVR).

### Visual interpretation

**Fig. 1:**
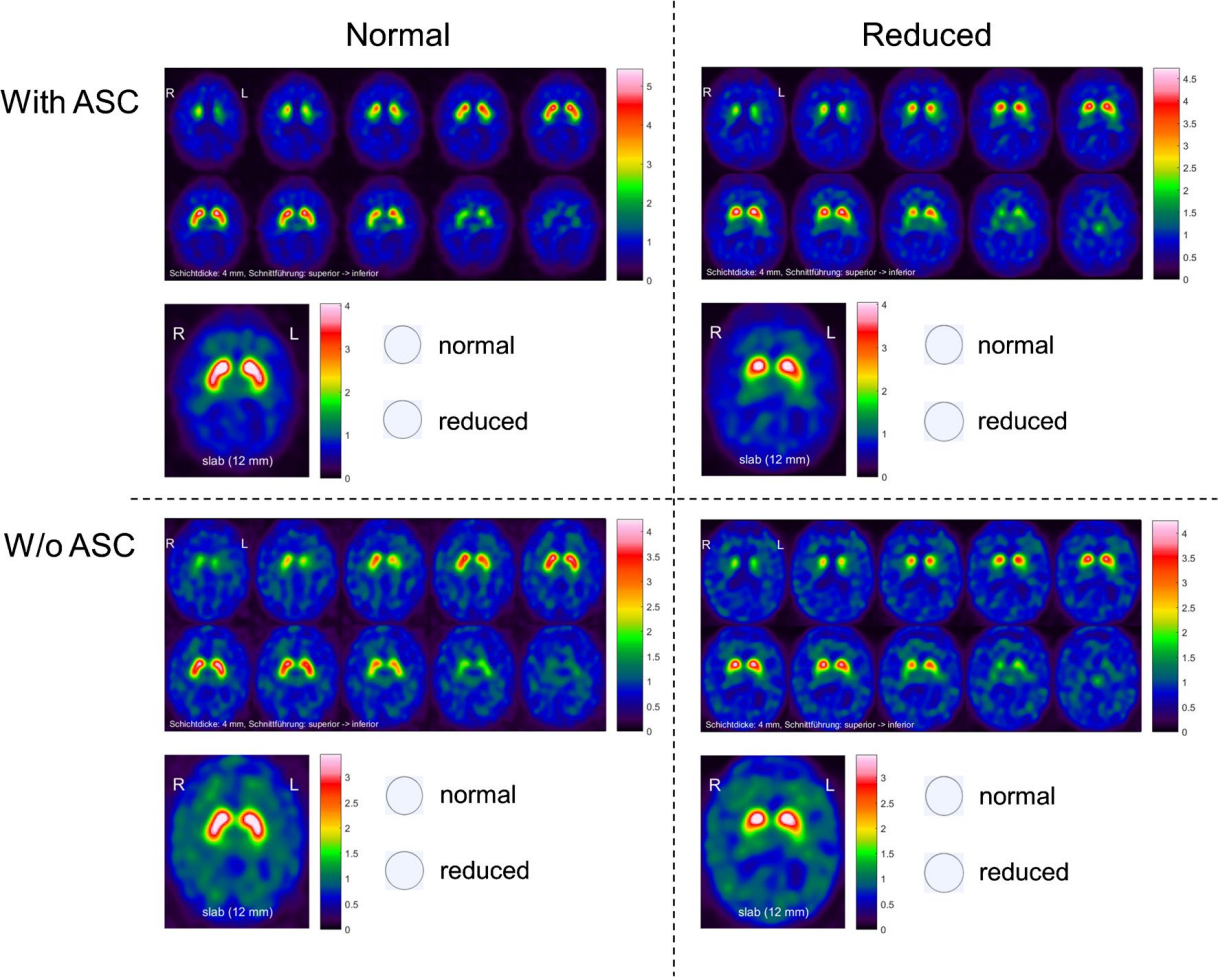
Standardized display used for visual interpretation of the DAT-SPECT images

A standardized display (Fig. 1) was used for the visual interpretation of the DAT-SPECT images, similar to the display used in everyday clinical routine at our site. The display comprised ten transversal DVR image slices of 4 mm thickness from the superior to the inferior edge of the striatum with the maximum of the color table individually scaled to the maximum intensity in the ten images. In addition, the display presented a transversal DVR image slab of 12 mm thickness through the center of the striatum with the maximum of the color table scaled to a fixed upper DVR threshold [34]. The DVR threshold had been optimized previously, separately for images with ASC (DVR = 4.05) and for images without ASC (DVR = 3.45), as inconsistent color mapping might lead to misinterpretation [19, 35].

Visual interpretation of the SPECT images was performed independently by three readers with different experience in the clinical reading of DAT-SPECT. Two readers had more than 10 years of experience (≥ 2000 cases); the third reader had no previous experience. The readers were blinded for all clinical data. They were asked for binary categorization of the SPECT images with respect to the presence of Parkinson-typical reduction (“reduced”) or the absence of Parkinson-typical reduction of striatal ^123^I-FP-CIT uptake (“normal”). Two training sessions, each with 48 randomly selected images with ASC and 48 randomly selected images without ASC, were performed independently by the three readers. The results of the training sessions were discussed among the 3 readers in order to harmonize visual interpretation between the readers.

For the main reading, four 1740-page PDF documents were created. Each PDF document presented the 1740 DAT-SPECT cases, one per page. Thus, a total of 6960 images were categorized by each reader. Two of the four PDF documents contained the same 1740 images with ASC, and the other two PDF documents contained the same 1740 images without ASC. The ordering of the 1740 images within a given PDF document was randomized using a random permutation of the integers from 1 to 1740 generated by the MATLAB routine “randperm.” This was performed separately for each of the four PDF documents (with different states of the random number generator to guarantee different randomization for each PDF document). The readers categorized the images by clicking “normal” or “reduced” in the PDF document (Fig. 1).

DAT-SPECT images with discrepant categorization between the two reading sessions were read a third time by the same reader to obtain an intra-readers consensus, separately for each reader.

### Semi-quantitative analysis

The ^123^I-FP-CIT SBR in left and right putamen was obtained by hottest voxels analysis of the stereotactically normalized DVR image using large unilateral putamen masks predefined in MNI space [36, 37]. The putamen masks were much bigger than the actual putamen volume to guarantee that all putaminal counts were included. The number of hottest voxels within a unilateral putamen mask to be averaged was fixed to a total volume of 10 ml. The putamen SBR was computed as the mean DVR in the hottest voxels minus 1. The minimum putamen SBR of both hemispheres was used for the analysis.

### Statistical analysis

Cross tables, Cohen’s kappa and the percentage of discrepant cases were used to characterize.

- Intra-reader variability of the visual categorization of the DAT-SPECT images, separately for the DAT-SPECT images with and without ASC and separately for each reader
- Between-reader variability of the visual categorization of the DAT-SPECT images (intra-reader consensus), separately for the DAT-SPECT images with and without ASC
- The impact of ASC on the visual categorization of the DAT-SPECT images (intra-reader consensus), separately for each reader and for the majority vote of the three readers
- The consistency between visual categorization (majority vote) and automatic SBR-based categorization of the DAT-SPECT images, separately for the DAT-SPECT images with and without ASC.

The impact of ASC on the discriminative power of the putamen SBR was tested as follows. The distribution of the putamen SBR was characterized by a histogram with 0.1 bin width. The resulting histogram was fitted by the sum of two Gaussians:

1 $$histogram\left(SBR\right)=A_1exp\;\left(-\frac{\left(SBR-M_1\right)^2}{{2SD}_1^2}\right)+A_2exp\;\left(-\frac{\left(SBR-M_2\right)^2}{{2SD}_2^2}\right)$$

where* A*_*1*_, *A*_*2*_ are the amplitudes, *M*_*1*_, *M*_*2*_ are the mean values, and *SD*_*1*_, *SD*_*2*_ are the standard deviations of the Gaussian functions. The MATLAB routine “fminsearch” with default parameter settings was used for this purpose.

The power of the SBR to differentiate between normal and reduced DAT-SPECT was estimated by the effect size *d* of the distance between the two Gaussians computed as the differences between the mean values scaled to the pooled standard deviation

2 $$d=\left({M}_{2}-{M}_{1}\right)/\sqrt{\frac{{SD}_{1}^{2}+{SD}_{2}^{2}}{2}}$$

The cutoff *c* for differentiation between normal and reduced SBR was selected halfway between *M*_*1*_ and *M*_*2*_ in units of standard deviations, that is

3 $$c=\left({{SD}_{2}M}_{1}+{{SD}_{1}M}_{2}\right)/\left({SD}_{1}+{SD}_{2}\right)$$

The putamen SBR was considered “reduced” if it was smaller than the cutoff *c*; it was considered “normal” if it was equal or larger than the cutoff *c*.

The histogram analysis was performed separately with and without ASC.

In order to assess a possible impact of the hardware setting on the discrimination between normal and reduced cases, the proportion of cases with discrepant categorization without versus with ASC was compared between the 4 different hardware settings, separately for visual interpretation and for the automatic categorization based on the semi-quantitative analyses. The chi-square test of the corresponding 4 × 2 cross tables (4 hardware settings × without versus with ASC) was used to test the observed differences for statistical significance.

## Results

### Intra- and between-reader agreement of the visual interpretation

Mean intra-readers kappa of the visual categorization of the DAT-SPECT images was 0.957 ± 0.012 (range 0.949–0.971) without ASC versus 0.956 ± 0.010 (0.949–0.967) with ASC (Table 1). The mean proportion of cases with discrepant categorization by the same reader was 2.13% ± 0.60% (range 1.44–2.53%) without ASC versus 2.20% ± 0.47% (1.67–2.53%) with ASC (Table 1).

**Table 1:** Intra-reader agreement across the two reading sessions for each of the three readers (R1–R3) and between-reader agreement for each pair of readers, separately without ASC and with ASC. Intra-reader agreement between the intra-reader consensus without ASC and the intra-reader consensus with ASC is also shown. Given are Cohen’s kappa (standard error in parenthesis) and the percentage (%) of discrepant cases (*n* = 1740)

Intra-reader	R1 (experienced)		R2 (experienced)		R3 (inexperienced)	
	Kappa	%	Kappa	%	Kappa	%
Without ASC	0.971 (0.006)	1.44%	0.952 (0.007)	2.41%	0.949 (0.008)	2.53%
With ASC	0.967 (0.006)	1.67%	0.952 (0.007)	2.41%	0.949 (0.008)	2.53%
Without vs. with ASC	0.978 (0.005)	1.09%	0.961 (0.007)	1.95%	0.961 (0.007)	1.95%
Between-reader	R1 vs. R2		R1 vs. R3		R2 vs. R3	
	Kappa	%	Kappa	%	Kappa	%
Without ASC	0.967 (0.006)	1.67%	0.924 (0.009)	3.79%	0.946 (0.008)	2.70%
With ASC	0.947 (0.008)	2.64%	0.921 (0.009)	3.97%	0.941 (0.008)	2.93%

The mean between-reader kappa of the visual categorization of the DAT-SPECT images was 0.946 ± 0.022 (range 0.924–0.967) without ASC versus 0.936 ± 0.014 (0.921–0.947) with ASC (Table 1). The mean proportion of cases with discrepant categorization between two readers was 2.72% ± 1.06% (range 1.67–3.79%) without ASC versus 3.18% ± 0.70% (2.64–3.97%) with ASC (Table 1).

### Impact of ASC on the visual interpretation

The mean without-versus-with-ASC kappa of the visual categorization of the DAT-SPECT images by the same reader was 0.967 ± 0.010 (range 0.961–0.978, Table 1). The mean proportion of cases with discrepant categorization of the same DAT-SPECT without and with ASC by the same reader was 1.66% ± 0.50% (range 1.09–1.95%). The without-versus-with-ASC kappa of the majority visual categorization of the DAT-SPECT images was 0.971 (standard error 0.006). A disagreement of the majority visual categorization of the same DAT-SPECT without and with ASC occurred in 25 of the 1740 DAT-SPECT (1.44%), with rather balanced disagreement in both directions (15/10 cases, Table 2).

**Table 2:** Visual and automatic SBR-based categorization of the DAT-SPECT images. With-versus-without-ASC cross table of the visual categorization of the DAT-SPECT images according to the majority of the three readers (intra-reader consensus) (top) and of the automatic categorization of the DAT-SPECT images based on the putamen SBR using the cutoff derived from the histogram analysis (Table 3). Given are the number and percentage of the cases (*n* = 1740)

Visual categorization		With ASC	
		Normal	Reduced
Without ASC	Normal	893 (51.3%)	15 (0.9%)
	Reduced	10 (0.6%)	822 (47.2%)
Automatic categorization		With ASC	
		Normal	Reduced
Without ASC	Normal	944 (54.3%)	25 (1.4%)
	Reduced	6 (0.3%)	765 (44.0%)

Among the 1740 DAT-SPECT, there was only one single case (0.06%) with discrepant categorization between without and with ASC by all 3 readers (Supplementary Fig. 1).

### Impact of ASC on semi-quantitative analysis

**Fig. 2:**
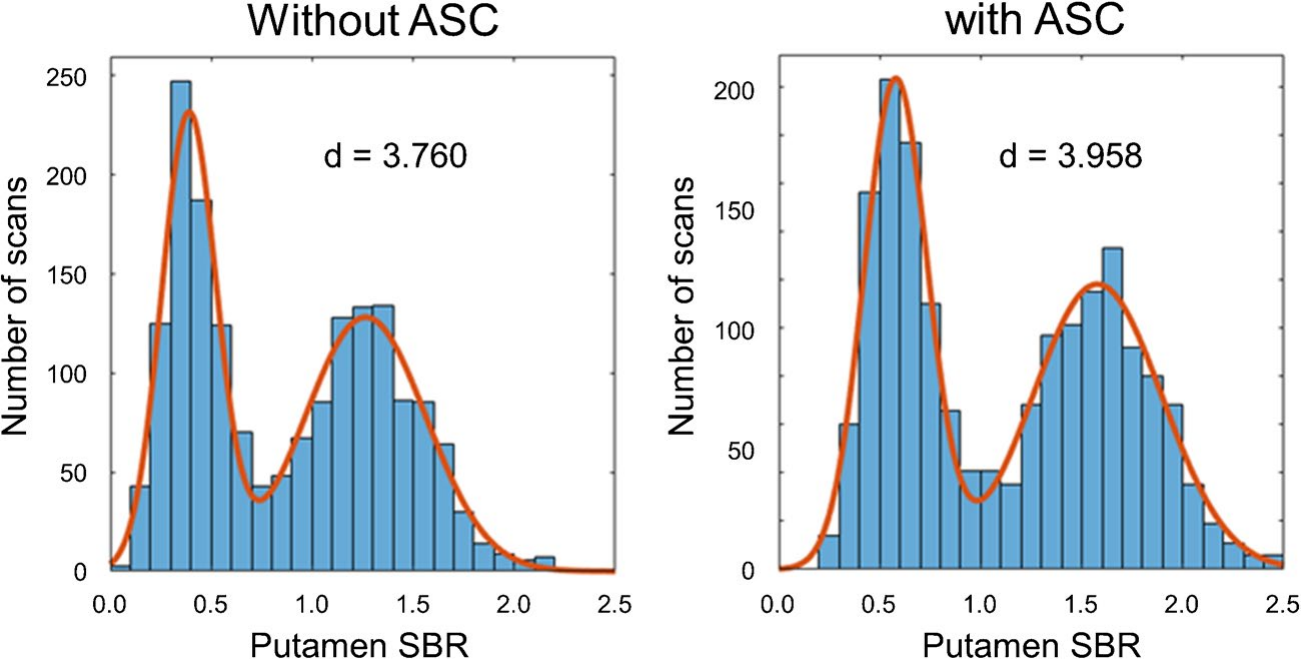
Histograms of the putamen SBR without ASC (left) and with ASC (right) in the sample of 1740 unselected DAT-SPECT from clinical routine. The fit by the sum of two Gaussians is indicated by a continuous line. The effect size *d* of the distance between the two Gaussian functions was computed according to Eq. (2)

The histograms of the putamen SBR without and with ASC and their fit by the sum of two Gaussians are shown in Fig. 2. The parameters obtained by the fit are given in Table 3. The effect size *d* of the distance between the two Gaussians was slightly larger with ASC than without ASC (3.958 versus 3.760). Cross tables of the automatic binary classification by the putamen SBR with versus without ASC using the cutoffs derived from the histogram analyses are given in Table 2. The without-versus-with-ASC kappa for the binary classification was 0.964 (standard error 0.006).

**Table 3:** Histogram analysis of the putamen SBR. Mean values *M*_*1*_, *M*_*2*_ and standard deviations *SD*_*1*_, *SD*_*2*_ from the fit of the histogram of the putamen SBR (*n* = 1740) by the sum of two Gaussians according to Eq. (1), effect size *d* of the distance between the two Gaussian functions according to Eq. (2), and cutoff *c* for the dichotomization of SBR as normal or reduced according to Eq. (3). The results are given separately for with and without ASC

	Reduced		Normal		Effect size	Cutoff
	*M*_*1*_	*SD*_*1*_	*M*_*2*_	*SD*_*2*_	*d*	*c*
Without ASC	0.389	0.135	1.264	0.300	3.760	0.661
With ASC	0.579	0.157	1.577	0.320	3.958	0.907

### Agreement of visual interpretation and automatic categorization

Cohen’s kappa between the automatic binary categorization based on the putamen SBR versus the visual binary categorization (majority vote) was 0.911 (standard error 0.010) without ASC and 0.918 (0.010) with ASC. The proportion of discrepant cases between the SBR-based categorization and visual categorization was 4.43% without ASC and 4.08% with ASC.

### Impact of the hardware setting

The proportion of cases with discrepant visual categorization (majority vote) between without versus with ASC was 1.70%, 2.04%, 0.67%, and 1.62% for the DAT-SPECT acquired with the Siemens e.cam with LEHR, the Siemens Symbia TruePoint with LEHR, the Siemens Symbia TruePoint with fan-beam, and the Mediso AnyScan Trio with LEHRHS, respectively. The proportion of cases with discrepant automatic categorization between without versus with ASC based on the putamen SBR using the cutoffs derived from the histogram analyses was 1.70%, 1.36%, 1.75%, and 2.08% for the DAT-SPECT acquired with the Siemens e.cam with LEHR, the Siemens Symbia TruePoint with LEHR, the Siemens Symbia TruePoint with fan-beam, and the Mediso AnyScan Trio with LEHRHS, respectively. The differences were not statistically significant, neither for the visual interpretation (*p* = 0.423) nor for the automatic categorization (*p* = 0.940).

## Discussion

The mean proportion of cases with discrepant categorization by the same reader between the two reading sessions was practically the same without and with ASC, about 2.2%. Thus, this proportion can be used as benchmark for the interpretation of ASC effects on the categorization of DAT-SPECT, a without-versus-with-ASC discrepancy of about 2.2% (or below) indicating “no relevant impact” of ASC.

Using this benchmark, the primary finding of the current study was the lack of a relevant impact of ASC on the binary categorization of DAT-SPECT both by visual interpretation and by conventional semi-quantitative SBR analysis, independent of the hardware setting. Regarding visual interpretation, the proportion of DAT-SPECT with discrepant categorization without versus with ASC in the whole sample by the same reader was 1.66% ± 0.50%, that is, even slightly below the benchmark of 2.2%. This is most likely explained by the use of the intra-reader consensus for the DAT-SPECT images without ASC and the intra-reader consensus of the DAT-SPECT images with ASC for the assessment of the intra-reader agreement between with and without ASC (stabilization by reaching a consensus). Regarding semi-quantitative analysis, the fit of the SBR histograms by the sum of two Gaussians revealed a slightly higher effect size *d* of the distance between the two Gaussians with ASC compared to without ASC. However, automatic categorization of the DAT-SPECT images based on the putamen SBR using the cutoffs derived from the histogram analysis resulted in discrepant categorization of the same DAT-SPECT with versus without ASC in only 1.78% of the cases, that is, also below the benchmark of 2.2%. Finally, addressing the consistency between visual interpretation and semi-quantitative analysis, the proportion of cases with discrepant visual and SBR-based categorization was very similar without and with ASC.

These findings indicate that ASC does not have a relevant impact on the detection of nigrostriatal degeneration in CUPS. This is in line with a previous study that reported no impact of ASC on the categorization of 73 DAT-SPECT from clinical routine by visual interpretation or semi-quantitative analyses [38]. Due to the small sample size, the authors of this previous study concluded that “larger studies are needed for the validation of the … result” [38]. In fact, post hoc analysis of the power that can be achieved by the McNemar test to detect a difference in the visual interpretation between with and without ASC at the one-sided 5% significance level assuming a proportion of discrepant cases of 10%, 5%, or 3% and twice as much reduced findings with ASC compared to without ASC among the discrepant cases (i.e., odds ratio = 2, for example, due to higher sensitivity for detection of nigrostriatal degeneration with ASC); the sample size of *n* = 73 provides only very low power of 11.6%, 3.4%, and 0.8%, respectively. The power of the 24 times larger sample of 1740 DAT-SPECT of the current study to detect an effect of ASC on the visual interpretation of DAT-SPECT at the one-sided 5% significance level assuming 10%, 5%, or 3% discrepant cases, and an odds ratio of 2 is 99.7%, 91.9% and 74.6%, respectively. Thus, the findings of the current study allow reasonable exclusion of a 3% difference in the visual interpretation between with and without ASC (assuming an odds ratio of 2), whereas the previous study did not provide sufficient power to reasonably exclude a 10% difference (at the same odds ratio).

There are several factors to explain the lack of a relevant impact of ASC on the detection of nigrostriatal degeneration. First, the effect of ASC is considerably smaller in DAT-SPECT with ^123^I-FP-CIT than in brain PET, due to the shorter path length to be covered by the single photon in SPECT compared to the total path length of both photons in PET, which overcompensates the higher attenuation coefficient of 159 keV photons from the decay of ^123^I compared to 511 keV photons from electron–positron annihilation. Second, neither the visual interpretation nor the conventional semi-quantitative analysis of DAT-SPECT is based on the signal of the striatum or striatal subregions in absolute terms but on the contrast of the striatum and its subregions relative to extrastriatal (reference) regions or relative to each other (e.g., the putamen-to-caudate ratio). The effects of photon attenuation and scatter cancel to some extent when considering contrasts or binding ratios, given that the extrastriatal (reference) regions are also affected by these physical effects, although to lesser extent than the striatum in the central part of the brain. Finally, the between-subject variability in striatal contrast due to between-subject variability of attenuation and scatter is much smaller than the 50% DAT loss threshold for the occurrence of motor symptoms secondary to nigrostriatal degeneration [39]. In the European Normal Control Database of DaTSCAN, ASC had only a very small effect on the between-subject variability of the striatal ^123^I-FP-CIT SBR (iteratively reconstructed images: 28% without ASC versus 26% with ASC) [10, 14].

The lack of a relevant impact of ASC supports institutions that consistently perform clinical DAT-SPECT without ASC. This eliminates limitations of ASC including the time required for and the risk of errors of the delineation of the outer contour of the head in case of uniform attenuation correction [32], as well as radiation exposure by the low-dose CT, additional scan time, and motion artifacts for CT-based ASC [18, 40]. Manual delineation of the head contour can be challenging in DAT-SPECT images with low scalp signal [14, 41]. Correction for scatter and septal penetration by the triple energy window method [42] adds statistical noise.

A secondary finding of this study was the good agreement of the visual categorization of the DAT-SPECT images between the two experienced readers (R1, R2; both ≥ 2000 cases), in line with previous studies [43]. Agreement of the inexperienced reader (R3) with the two experienced readers was somewhat lower, suggesting that the visual categorization of DAT-SPECT is affected by reader experience. Inspection of the cross tables of the visual categorization between the inexperienced reader R3 and the experienced reader R1 (not shown) revealed that all discrepant cases were categorized as normal by the experienced reader R1 and as reduced by the inexperienced reader R3. This suggests a too sensitive reading of the DAT-SPECT images by the inexperienced reader, that is, misinterpretation of minor left–right asymmetry and/or minor reduction of the striatal signal (below the 50% threshold for the occurrence of motor symptoms) as indication of nigrostriatal degeneration.

Partial volume effects, caused by the limited spatial resolution of SPECT and mixing of tissues in finite-sized voxels, result in strong underestimation of the conventional putamen SBR in DAT-SPECT even when attenuation and scatter correction is performed [17, 19, 44, 45]. Multiple algorithms have been proposed for the correction of partial volume effects [46], some of which require only the SPECT image itself [47, 48] whereas potentially more accurate methods make use of detailed anatomical information provided by a high-resolution MRI scan from the same subject [49, 50]. Among the correction methods that do not require an individual MRI, the “Southampton” method is particularly easy to implement in clinical practice, since it based on conventional region-of-interest analysis of the reconstructed image [29, 48, 51, 52]. The Southampton method accounts for partial volume effects by using the total number of striatal counts rather than striatal count density to estimate the SBR. The Southampton method cannot be applied on a voxel-by-voxel base and, therefore, does not affect visual interpretation of DAT-SPECT.

There are early signs of Parkinson’s disease such as loss of smell and rapid eye movement sleep and behavior disorder that precede motor symptoms. As soon as disease-modifying drugs for the treatment of Parkinson’s disease will be available, it will be important to detect Parkinson’s disease also in these early stages, when the loss of (unilateral) putaminal DAT is considerably below the 50% threshold for motor symptoms. The current results do not allow conclusions about the impact of ASC on the diagnostic performance of DAT-SPECT in these pre-motor disease stages.

Another limitation of the current study is that there was no gold standard diagnosis available such as clinical diagnosis by a movement disorder specialist ≥ 2 years after DAT-SPECT. Thus, conventional performance measures such as sensitivity and specificity for the identification of neurodegenerative parkinsonian syndromes could not be determined. However, this does not affect the findings with respect to the impact of ASC on semi-quantitative analysis and visual interpretation of DAT-SPECT, since there was no significant effect of ASC which should have been interpreted as beneficial or detrimental.

Finally, uniform attenuation correction was used in the current study, in line with common procedure guidelines [22, 23, 40]. The EANM practice guideline/SNMMI procedure standard for dopaminergic imaging in parkinsonian syndrome states that “attenuation correction with a uniform attenuation map can be used effectively with an appropriate linear attenuation correction coefficient for ^123^I and careful contouring of the head/scalp” [40]. However, iterative reconstruction algorithms can also use non-uniform attenuation maps, for example, attenuation maps derived from a low-dose CT when a SPECT/CT hybrid system is used (CT-based attenuation correction, CTAC). CTAC has been reported to improve the performance of voxel-based statistical testing to detect regional perfusion changes in cerebral blood flow SPECT images compared to uniform attenuation correction [53]. However, available studies (all with rather small sample size) do not suggest that CTAC is superior to uniform attenuation correction with respect to the detection of nigrostriatal degeneration using DAT-SPECT [19, 54, 55]. The EANM practice guideline/SNMMI procedure standard therefore states that “in the few available studies comparing data obtained using CTAC with data obtained using uniform attenuation correction, minor differences were observed, without impact on diagnostic performance.” However, the findings of the current study do not rule out that more accurate attenuation correction methods such as CTAC might have a relevant impact on the clinical utility of DAT-SPECT to detect nigrostriatal degeneration in patients with a clinically uncertain parkinsonian syndrome.

In conclusion, the present study provides strong evidence that ASC with uniform attenuation correction and simulation-based scatter correction has no relevant impact on the binary categorization of DAT-SPECT images as normal or reduced by visual interpretation or conventional semi-quantitative analysis. However, it is highly recommended to be strictly consistent with respect to the use of ASC (“always or never”), since constant switching between with and without ASC causes additional variability of no interest which complicates the interpretation of DAT-SPECT, particularly when using semi-quantitative analyses.

### Supplementary Information

Below is the link to the electronic supplementary material.

Supplementary file1 (DOCX 1144 KB)

## Data Availability

The data generated during the current study (visual interpretation, semi-quantitative data) are available from the corresponding author on reasonable request.

## Abbreviations

^123^I-FP-CIT: N-ω-fluoropropyl-2β-carbomethoxy-3β-(4-I-123-iodophenyl)nortropane
*A*: Amplitude
ASC: Attenuation and scatter correction
*c*: Cutoff
CTAC: CT-based attenuation correction
CUPS: Clinically uncertain parkinsonian syndrome
*d*: Effect size
DAT: Dopamine transporter
DVR: Distribution volume ratio
*M*: Mean value
MNI: Montreal Neurological Institute
SBR: Specific binding ratio
*SD*: Standard deviation
SPECT: Single photon emission computed tomography
SPM: Statistical Parametric Mapping
