# Supplementary material for: No impact of attenuation and scatter correction on the interpretation of dopamine transporter SPECT in patients with clinically uncertain parkinsonian syndrome
Source: Eur J Nucl Med Mol Imaging. 2023 Jun 17;50(11):3302–12. doi: 10.1007/s00259-023-06293-2 (PMC10541531; doi:10.1007/s00259-023-06293-2)
Supplement: Supplementary file 1 — Supplementary file1 (DOCX 1144 KB) [file 259_2023_6293_MOESM1_ESM.docx]

**Supplementary Tab. 1** SPECT acquisition and reconstruction parameters

|  | Siemens e.cam with LEHR | Siemens Symbia TruePoint with LEHR | Siemens Symbia TruePoint with fan-beam | Mediso AnyScan Trio with LEHRHS (dual head mode) |
| --- | --- | --- | --- | --- |
| # scans | 704 | 147 | 457 | 432 |
| Acquisition parameters | | | | |
| # views | 128 | 120 | 120 | 120 |
| Scan arc [°] | 180 | 180 | 180 | 180 |
| Angular step [°] | 2.81 | 3 | 3 | 3 |
| Matrix | 128x128 | 128x128 | 128x128 | 128x128 |
| Pixel size [mm^2^] | 4.80x4.80 | 3.90x3.90 | 3.90x3.90 | 2.43x2.43 |
| Energy window [keV] | 147-171 | 147-171 | 147-171 | 143-175 |
| Total net duration [min] | 32-41 | 30-40 | 40 | 40 |
| Collimator parameters for resolution recovery during reconstruction | | | | |
| Hole diameter [cm] | 0.111 | 0.111 | 0.153 | 0.120 |
| Hole length [cm] | 2.405 | 2.405 | 3.500 | 2.400 |
| Detector resolution [cm] | 0.38 | 0.38 | 0.38 | 0.36 |
| Radius of rotation offset [cm] | 3.205 | 3.205 | 4.300 | 3.200 |


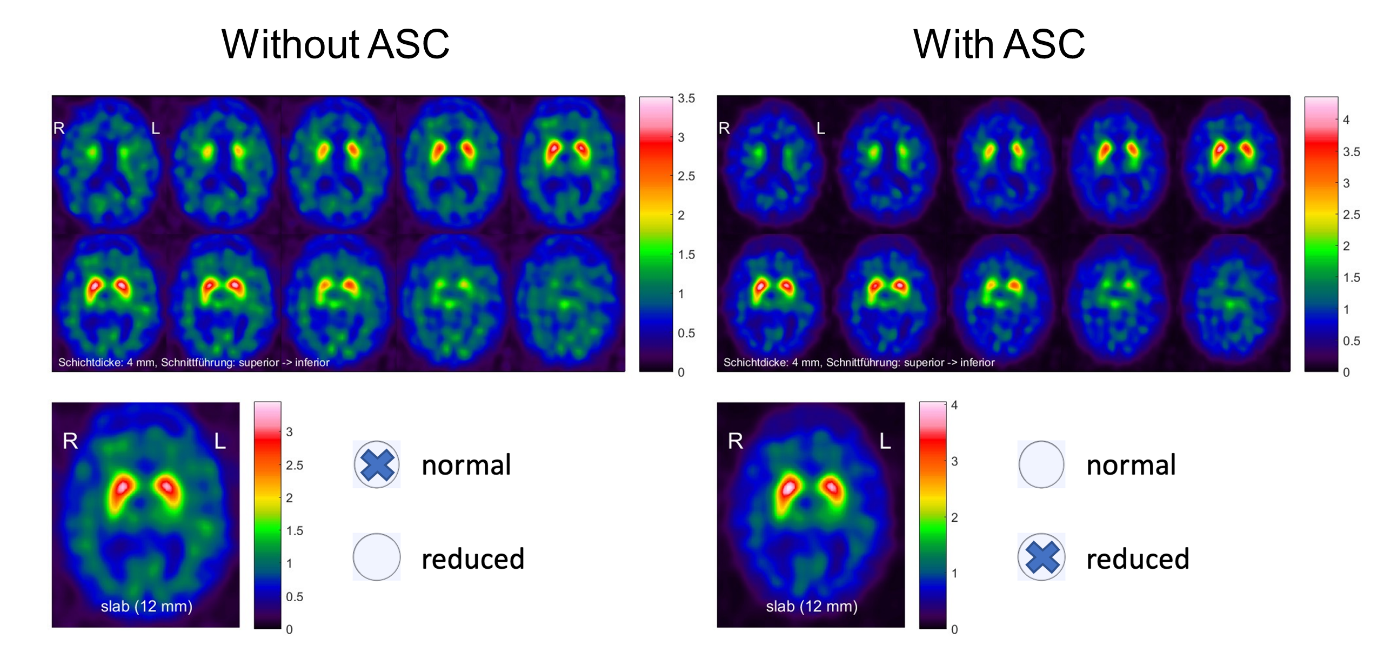


**Supplementary Fig. 1** DAT-SPECT of an 82 years old man with discrepant categorization: the images without ASC (left) were categorized as normal by all three readers, the images with ASC (right) were categorized as reduced by all three readers
